# *In vitro* assessment of chemical surface treatments on the shear bond strength of metal orthodontic brackets to CAD/CAM provisional materials

**DOI:** 10.3389/fdmed.2024.1494484

**Published:** 2024-12-12

**Authors:** Abdulaziz A. Alzaid, Khalid K. Alanazi, Maha N. Alharbi, Lulu A. Alyahya, Hatem Alqarni, Mohammed Alsaloum, Hayam Alfallaj, Ghada S. Alotaibi

**Affiliations:** ^1^ Restorative and Prosthetic Dental Sciences Department, College of Dentistry, King Saud Bin Abdulaziz University for Health Sciences, Riyadh, Saudi Arabia; ^2^ King Abdullah International Medical Research Center, Riyadh, Saudi Arabia; ^3^ Conservative Dental Science Department, College of Dentistry, Prince Sattam bin Abdulaziz University, Al-kharj, Saudi Arabia; ^4^ College of Dentistry, King Saud Bin Abdulaziz University for Health Sciences, Riyadh, Saudi Arabia; ^5^ King Abdulaziz Medical City, Ministry of the National Guard—Health Affairs, Riyadh, Saudi Arabia

**Keywords:** adhesive performance, dental materials, orthodontic bonding, surface modification, temporary restorations

## Abstract

**Introduction:**

The growing demand for orthodontic treatment in patients irrespective of age highlights the need for effective bonding of brackets to provisional crowns (PCs).

**Aims and objectives:**

This study evaluates the shear bond strength (SBS) of orthodontic brackets to 3D-printed and milled PC materials, comparing the effects of hydrofluoric acid (HFA) and phosphoric acid (PA) etching.

**Materials and methods:**

Forty cylinders were fabricated using a 3D printer with hybrid resin, and forty were milled from cross-linked polymethyl methacrylate (PMMA) resin. Stainless steel brackets were bonded with light-cured composite resin. Twenty specimens from each group were treated with 9.5% HFA, while the rest of the specimens received 37% PA. Post-bonding, specimens underwent thermocycling and were examined with SEM. SBS testing followed ISO/TS 11405-2015 guidelines. The failure patterns and bond interface were assessed by the Adhesive Remnant Index (ARI) and scanning electron microscopy (SEM). Data was analyzed using ANOVA, Tukey's test.

**Results:**

In 3D-printed materials, HFA etching yielded a significantly higher bond strength (12.59 ± 2.64 MPa) than PA etching (7.77 ± 0.83 MPa). The bond strength was inferior in milled materials: HFA (5.98 ± 0.59 MPa) and PA (5.66 ± 0.65 MPa) with no significant difference between both surface treatments. When each material was evaluated separately, a significant difference in SBS was found for surface treatments in 3D-printed materials (*p* < 0.001) but not for milled materials (*p* = 0.916). ARI scores showed greater adhesive retention in 3D-printed specimens, particularly those treated with HFA. SEM revealed smoother surfaces in 3D-printed specimens compared to rougher surfaces in milled specimens.

**Conclusion:**

HFA etching improves SBS in 3D-printed PC, while in milled materials, the choice of etching agent has minimal effect.

## Introduction

The increased demand for orthodontic treatment in adults has highlighted the importance of proper bonding of brackets to the surfaces of crowns, whether permanent or provisional. Orthodontic forces frequently need to be applied to teeth restored with provisional restorations. In such cases, adequate bonding of brackets to provisional crowns (PCs) is crucial for the effective application of orthodontic forces. Debonding during treatment can lead to complications such as delays in treatment completion and the risk of aspiration of brackets (1, 2).

Surface treatment of PC has been shown to improve the bonding of orthodontic brackets. Various surface treatments are applied to enhance surface roughness and bonding surface area. Mechanical methods include sandblasting and surface grinding with silicon carbide paper or a diamond bur (3). Chemical methods involve hydrofluoric acid (HFA) etching and the application of a silane primer (4). Combining mechanical and chemical surface treatments has been found to have a significant impact on bonding strength (2, 5, 6).

Advances in digital technology have led to the development of subtractive manufacturing (SM), such as milling, and additive manufacturing (AM), like 3D-printing, for producing dental prosthetics (7). 3D- printing is a manufacturing process that creates structures by building them up in incremental layers. Subtractive manufacturing technology, in the form of CAD/CAM milling, involves removing material from a prefabricated block or disc. 3D- printing offers advantages such as the ability to create complex restorations with intricate internal geometries and reduced material waste (8, 9). While milled materials generally have higher density and fewer defects, 3D-printed provisionals often exhibit superior mechanical properties, excluding microhardness, toughness, and resilience (10, 11). Various additive manufacturing methods are used in dentistry, with the most common being stereolithography (SLA), selective laser sintering (SLS), fused deposition modeling (FDM), digital light processing (DLP), PolyJet, and bioprinting.

This study presents a unique investigation into the effect of HFA surface treatment on the bond strength of orthodontic brackets, comparing its impact on both 3D-printed and milled PC materials. Unlike previous studies that have typically focused on conventional materials or a single method of fabrication, this research introduces a novel comparison between different types of PC materials fabricated through advanced techniques (CAD/CAM 3D-printing and milling).

## Study design

The study aims to evaluate and compare the effect of hydrofluoric acid (HFA) and phosphoric acid (PA) etching on the shear bond strength (SBS) of orthodontic brackets to 3D-printed and milled PC materials.

Objectives of the study:

1. To assess the SBS of orthodontic brackets bonded to 3D-printed PCs using HFA etching.
2. To assess the SBS of orthodontic brackets bonded to milled PCs using HFA etching.
3. To compare the effectiveness of HFA etching vs. PA etching in improving SBS and to determine whether 3D-printed or milled PC materials provide superior SBS for orthodontic bracket bonding.

Null hypothesis (H_0_): No significant difference exists in the SBS of orthodontic brackets bonded to 3D-printed and milled PCs, regardless of the etching method used (HFA or PA).

## Material and methods

**Figure 1:**
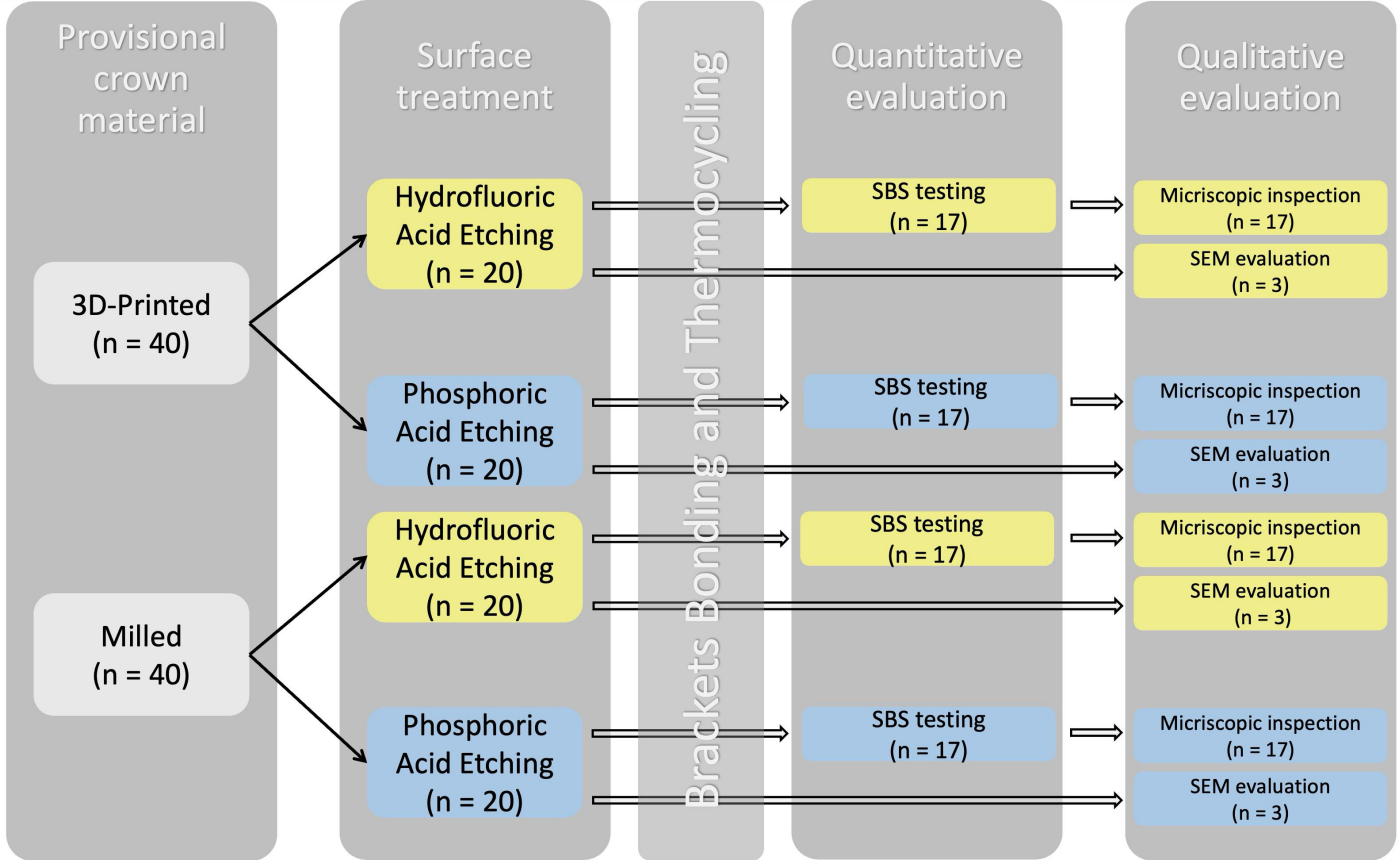
Flowchart showing the study design.

For the purpose of this study, specimens were designed as cylinders measuring 15 mm in diameter and 15 mm in height using 3D designing software [Meshmixer (Version 3.5); Autodesk, San Francisco, CA, USA]. A Standard Triangle Language (STL) file was created to fabricate all specimens tested in this study. Following completion of the design the STL file was used to produce the 3D-printed and milled PC materials to be tested in this study. Forty specimens were produced for each PC material, of which 20 specimens were randomly selected to be surface treated with either HFA or PA (*n* = 20) (Table 1). Then, for each surface treatment, 17 of the 20 specimens were used for quantitative testing and subsequent qualitative assessment with digital microscopy, with a total sample size of 68 (Figure 1). The other 3 specimens were used for qualitative assessment with scanning electron microscopy (SEM), with a total sample size of 12. For quantitative testing, the sample size per group was calculated to be 17, using G-power software (G * Power 3.1.9.7, Dusseldorf, Germany) (12) with an effect size of 0.996, power 0.80 and *α* 0.05. PC materials tested are listed in Table 2.

**Table 1:** Showing samples group distribution.

Surface treatment	3D printed PC material	Milled PC material
Hydrofluoric acid (HFA) etching	3D-HFA (*n* = 20)	M-HFA (*n* = 20)
Phosphoric acid (PA) etching	3D-PA (*n* = 20)	M-PA (*n* = 20)

**Table 2:** The composition and manufacturer of the 3D-printed and milled PC material used in the study.

Material	Manufacturer	Ref no./Lot no.	Composition	Technology	Indication
Detax Freeprint Temp	DETAX GmbH, Ettlingen, Germany	04063/251210	Monomers and oligomers/polymers encapped with a (meth-) acrylate group; - <5% of modified silicic acids; - Isopropylidenediphenol peg-2 dimethacrylate (45-<60%); - 7,7,9 (or 7,9,9)-trimethyl-4,13-dioxo-3,14-dioxa-5,12-diaza- hexadecane-1,16-diyl bismethacrylate (30-<35%); - 1,6-hexanediol dimethacrilate (1-<5%) - 2-hydroxyethyl methacrylate (1-<5%) - Diphenyl (2,4,6-trimethylbenzoyl) phosphine oxide (1-<5%)—Hydroxy propyl methacrylate (1-<5%) - Phenyl bis (2,4,6-trimethylbenzoyl)-phosphine oxide (<1%) Ceramic micro-filler (lower content)	DLP 3D printing	Temporary crowns and bridges and implant- supported restorations
Telio CAD	Ivoclar Vivadent, Schaan, Liechtenstein	686310/YBB1TX	99.5 wt.% PMMA, no fillers, pigment (<1 wt.%)	Milling	Temporary crowns and bridges and implant- supported restorations

Forty cylinders were fabricated using a Digital Light Processing (DLP) 3D printer (Asiga Max UV, Alexandria, Australia) composed of micro-filled hybrid provisional resin material (FREEPRINT® TEMP NM; Detax, Ettlingen, Germany) (Table 2). 3D-printed specimens were printed in a 45 degree orientation, a layer thickness of 50 μm and 385 nm wavelength. Then, specimens were carefully removed using a scraper and pre-cleaned in an ultrasonic bath of 99,9% isopropyl alcohol for 4 min. Then, specimens went through a cleaning cycle in a different ultrasonic bath with fresh 99,9% isopropyl alcohol for 6 min. Following cleaning, specimens were dried with compressed air and support structures were removed using Low speed rotary instrument. Final polymarization was completed using a Xenon flash light curing unit (2 × 2,000 flashes Nitrogen, 300–700 nm, Otoflash G171-N2, NK Optik GmbH Baierbrunn, Germany) under protective gas for 7 min.

Additionally, another forty cylinders with the same dimensions described above were wet milled from provisional material composed of cross-linked polymethyl methacrylate (Telio CAD, Ivoclar Vivadent, Schaan, Liechtenstein) (Table 2) using a 5-axis milling machine (Ceramill Motion 2, Amann Girrbach AG, Austria). Milling tools with diameters of 1 and 2.5 mm (Roto RFID, Amann Girrbach AG, Austria) were used to achieve the desired cylindrical shape. Cross-cut tungsten carbide burs (1958-012 Jet Tungsten Carbide Bur, Kerr, Kloten) were used to separate the specimens from the disc and smooth out the attachment points.

Following completion of the manufacturing process, both 3D-printed and milled specimens were steam cleaned and visually inspected for any defects. Then, all specimens were polished under water cooling using a sequence of silicon carbide (SiC) papers of decreasing grit (500–800–1,200–2,000–4,000, Struers, Ballerup, Denmark). A guide, with a circular central opening measuring 6 mm in diameter, was designed and 3D printed in a similar to the process described earlier. The purpose of the guide is to seat on the cylinders outlining the area to be surface treated in the middle of the cylindrical specimens surface where the brackets will be bonded and aid the bonding process.

Twenty specimens from both the 3D-printed and milled provisional materials were treated with 9.5% HFA etching (Bisco, Schaumburg, IL, U.S.A) for 30 s, while the remaining 20 specimens were treated with 37% PA (DentKist Inc. Gunpo, Gyeonggi-do, Korea) for 30 s.

Following completion of surface treatments, primer (Transbond XT Primer; 3M, St. Paul, MN, USA) was applied to the surface of each provisional material cylinder and air dried for 5 s to form an even layer. Then, stainless steel maxillary central incisor brackets (3M Unitek, St. Paul, MN, USA) were bonded to the provisional material cylinders using a light-cured composite resin (Transbond XT, 3M Unitek, St. Paul, MN, USA). The excess adhesive was removed with a dental explorer, and the adhesive was light-cured (3M Elipar, St. Paul, MN, USA) for a total of 10 s–5 s on each of the mesial and distal sides—as recommended by the manufacturer. Then, specimens were rinsed with water spray for 20 s and dried with compressed air for another 20 s.

After the brackets were bonded, all specimens were stored in distilled water at 37°C for 24 h. To simulate the oral cavity environment, thermocycling (Thermocycler: Huber, SD Mechatronik Thermocycler, Germany) was done by subjecting the specimens to 5,000 thermal cycles between 5°C and 55°C, with a dwell time of 30 s in an artificial saliva solution artificial (ASTM E2720-16 and ASTM E2721-16 with stabilized Mucin, Pickering Labs, Mountain View, CA, USA).

Following thermocycling, three specimens from each group were randomly selected for bonding interface evaluation using SEM (JEOL 6610LV series SEM, JEOL Ltd., Tokyo, Japan). Specimens were embedded in acrylic resin and sectioned perpendicular to the bracket-adhesive interface. The specimen was polished using 1 μm diamond slurry before SEM inspection. The analyses were conducted in both secondary electron (SE) and backscattered electron (BSE) modes at magnifications of 30×, 100×, and 500×, with an accelerating voltage of 15 kV.

The SBS test was conducted on 17 specimens from each group following ISO/TS 11405-2015 guidelines (13). A custom apparatus was designed to secure the cylinders onto a universal testing machine (5965: Instron, Canton, MA, USA) to apply force parallel to the bonding interface at a speed of 0.5 mm/min until bond failure occurred. The maximum force applied was recorded.

To assess the bond failure interface, the de-bonded bracket bases and provisional material cylinders (*n* = 17 per group) were visually examined, and images were taken using a digital microscope (Digital Microscope, KH-7700, Hirox-USA, Inc., Hackensack, NJ, USA) at 50 × magnification. Bond failure was classified according to the Adhesive Remnant Index (ARI) score, which evaluates the residual adhesive on the bonded area of the cylinder (Table 3). All measurements from the images were done using image editing software (2D Image File Viewing Software, KH-7700, Ver.2.10c, ©Hirox Co. Ltd. 2010, Hackensack, NJ, USA).

**Table 3:** Scoring of adhesive remnant index (ARI).

Description	Score
No adhesive left on the cylinder	0
Less than 50% of adhesive left on the cylinder	1
More than 50% of adhesive left on the cylinder	2
100% of adhesive left on the cylinder with a distinct impression of the bracket mesh	3

## Statistical analysis

The statistical analysis was performed using SPSS software (IBM, Ver: 28.0, Armonk, NY: IBM Corp, Chicago, IL, USA) with a significance level set at 0.05. Two-way Analysis of Variance (ANOVA) was used to determine the effect of each variable (provisional material and surface treatment) on the SBS. One-way Analysis of Variance (ANOVA) was used to detect any significant difference in SBS between the groups, followed by *post hoc* Tukey's test. For the qualitative analysis, two different observers evaluated the ARI index and SEM analysis.

## Results

Two-way ANOVA was used to determine the effect of each variable (provisional material and surface treatment) on the SBS. Statistically significant difference was found for both provisional material and surface treatment (*p* < 0.001) on SBS of orthodontic brackets to PC material.

The mean values and standard deviations for different groups, comparing the effects of surface treatment (HFA and PA etching) on 3D-printed and milled materials are listed in Table 4. Mean de-bond load values decreased in the following order: 3D-HFA > 3D-PA > M-HFA > M PA. In 3D-printed material, HFA etched group has the highest mean de-bond load of 151.02 ± 31.75 N, ranging from 78.86 N–221.05 N. This indicates that HFA etching improves the strength of 3D-printed materials. PA etched group's mean de-bond load drops to 93.26 ± 10.02 N, with a range of 70.00 N–107.56 N. This suggests that PA etching is less effective in enhancing the bond strength of brackets to 3D-printed materials compared to HFA. In milled materials, the HFA etched group has a mean de-bond load of 71.81 ± 7.14 N, with a range of 59.24 N–86.45 N. The de-bond load is notably lower compared to 3D-printed materials with the same treatment. PA etched specimens had mean de-bond load slightly lower at 67.93 ± 7.91 N, ranging from 51.81 N–83.36 N.

**Table 4:** Showing means and standard deviation for all groups and statistical significance for all groups.

Provisional material	Surface treatment	Load	Load	SBS	SBS
		(N)	(N)	(MPa)	(MPa)
		Mean ± (SD)	Min–Max	Mean ± (SD)*	Min–Max
3D-printed	Hydrofluoric Acid Etching (3D-HFA)	151.02 ± (31.75)	78.86–221.05	12.59 ± (2.64)	6.58–18.44
	Phosphoric Acid Etching (3D-PA)	93.25 ± (10.01)	70.00–107.56	7.77 ± (0.83)	5.84–8.97
Milled	Hydrofluoric Acid Etching (M-HFA)	71.81 ± (7.14)	59.24–86.45	5.98 ± (0.59)^a^	4.94–7.21
	Phosphoric Acid Etching (M-PA)	67.92 ± (7.90)	51.81–83.36	5.66 ± (0.65)^a^	4.32–6.95

No statistically significant difference for groups with the same superscript letter (a).

* Tukey HSD test showed no statistically significant difference for groups with the same superscript letter.

One-way ANOVA revealed significant difference between groups tested (*p* < 0.001). Tukey HSD test showed statistically significant difference (*p* < 0.05) between all material and surface treatment combinations comparisons except for milled provisional material with both surface treatments (*p* = 0.916).

The Mean SBS Values of 3D-printed materials shows that HFA etching (mean = 12.59 ± 2.64 MPa) resulted in significantly higher SBS values (*p* < 0.001) compared to PA etching (mean = 7.77 ± 0.83 MPa). Further, when compared to HFA (mean = 5.98 ± 0.59 MPa) and PA (mean = 5.66 ± 0.65 MPa) etched milled material, HFA etched 3D-printed materials showed significantly higher SBS values (*p* < 0.001). For PA etched 3D-printed material, there was a statistically significant difference with both HFA (*p* = 0.004) and PA etched milled material (*p* < 0.001). In milled materials, no significant difference (*p* = 0.916) in SBS was observed between HFA etching (mean = 5.98 ± 0.59 MPa) and PA etching (mean = 5.66 ± 0.65 MPa).

**Figure 2:**
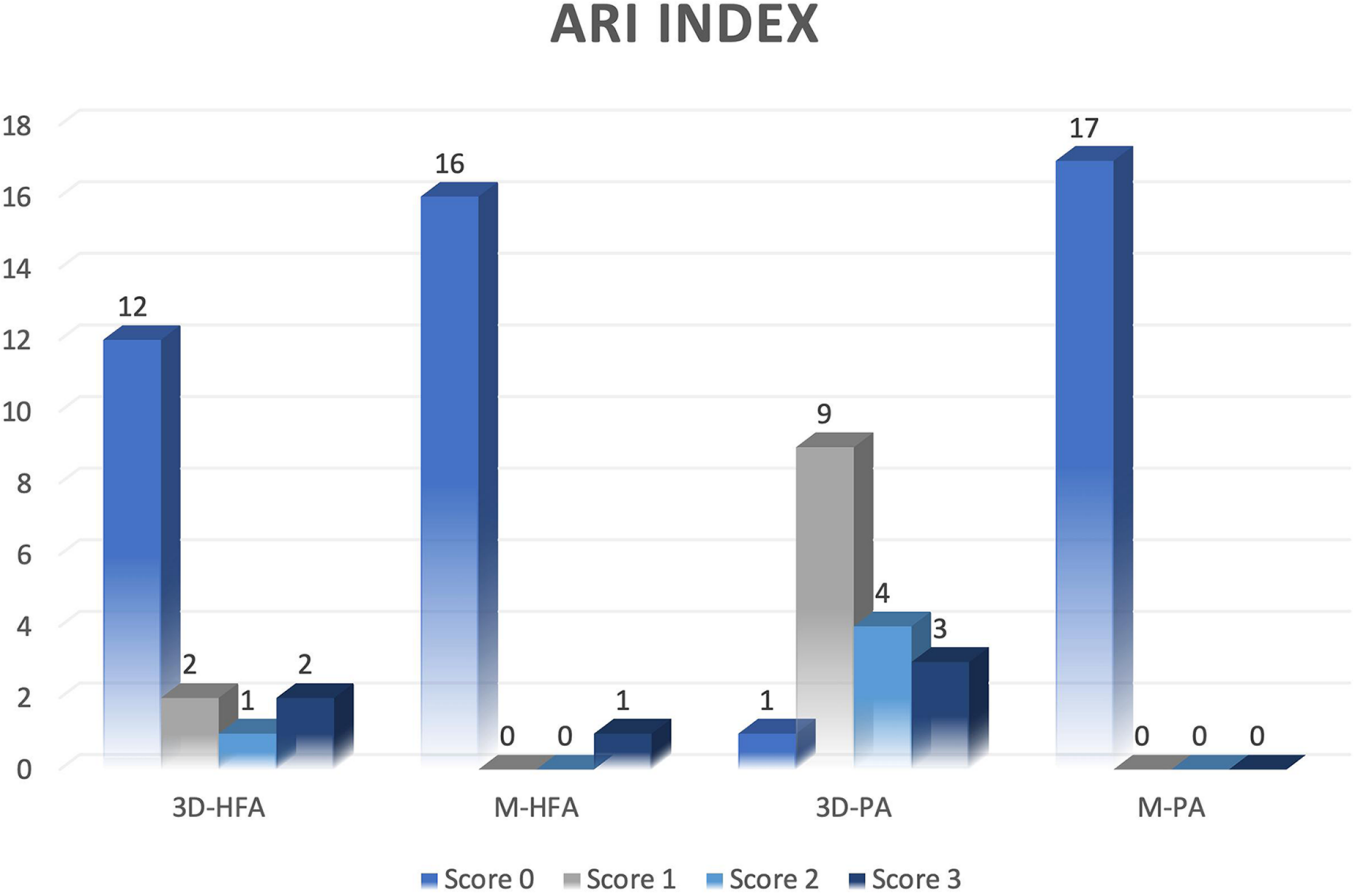
ARI index scores of the 17 specimens. (3D-HFA: Group 3D-printed with hydrofluoric acid etching, Group M-HFA: milled with hydrofluoric acid etching, Group 3D-PA: 3D-printed with phosphoric acid etching, Group M-PA: milled with phosphoric acid etching.

The ARI index was employed to evaluate the bond failure characteristics. Upon examination under a digital microscope, it was observed that the 3D-printed HFA-treated cylinders had a higher occurrence of scores 2 or 3 (3 specimens), while the milled specimens predominantly exhibited a score of 0, with only one exception (Figure 2). For the 3D-printed PA treated specimens, the majority had a score of 1 (9 specimens), whereas all the milled PA specimens showed a score of 0. In the milled control group, all specimens displayed adhesive fracture (ARI score 0) between the provisional material and adhesive indicating that no adhesive was left on the sample surfaces. Cohesive failure between the bracket and adhesive was less frequent in milled provisional materials compared to 3D-printed specimens.

**Figure 3:**
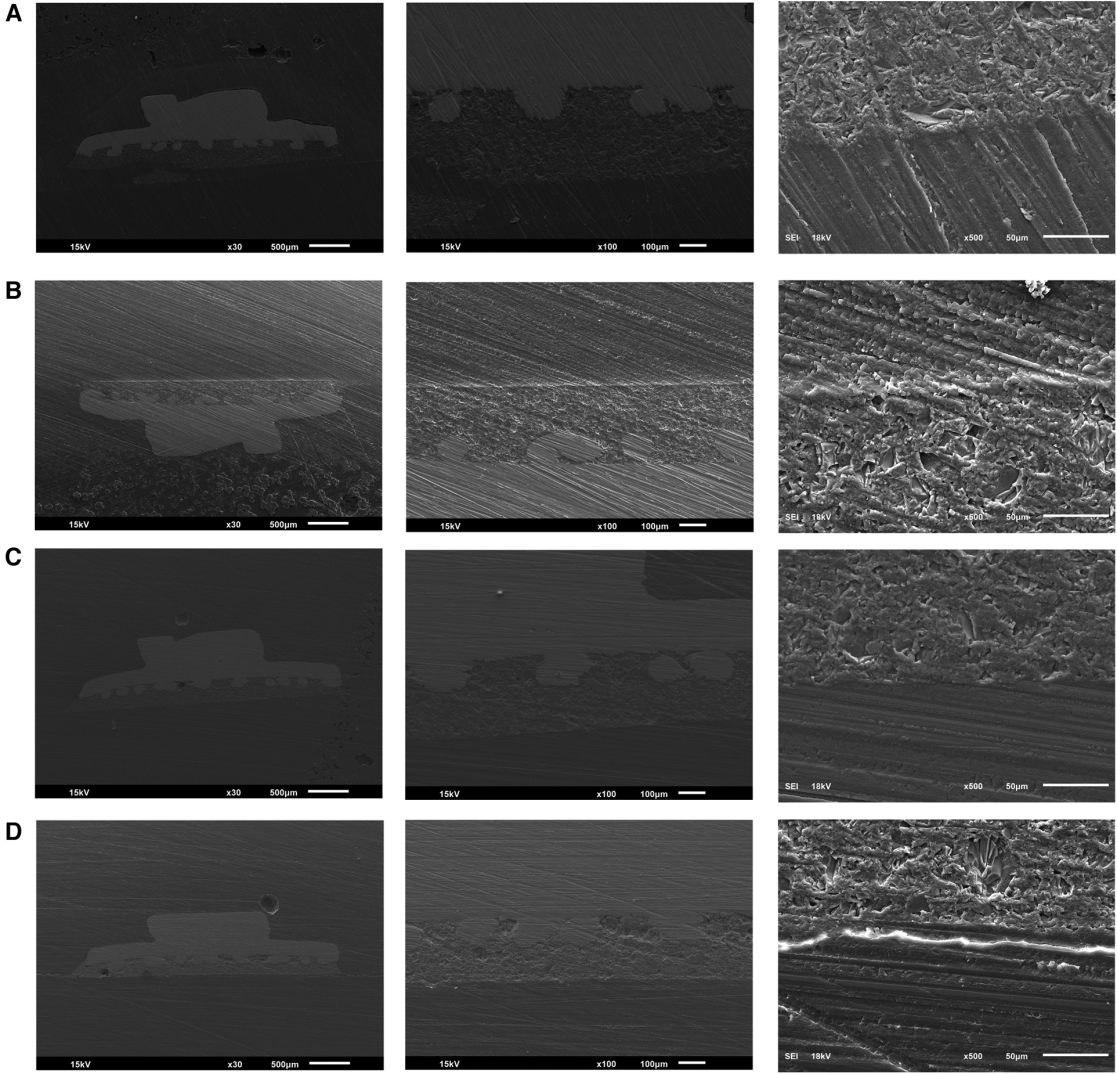
**(A)** SEM image of 3-D printed HFA treated specimens. **(B)** SEM image of 3-D printed PA-treated specimens shows the least surface irregularities. **(C)** SEM image of milled HFA-treated specimens shows the highest irregularities. **(D)** SEM image of milled PA-treated specimens. SEM images of 3D-printed and milled specimens treated with HFA and PA showing the bonding interface in three magnifications 30×, 100×, and 500×.

SEM images analysis in three magnifications 30×, 100×, and 500×, revealed significant differences between 3D-printed and milled specimens (Figure 3). The 3D-printed specimens exhibited smoother surfaces with well-defined etch pits and a clear grain structure. In contrast, the milled specimens displayed rougher surfaces with larger, more irregular features. Comparing the HFA and PA treatments, the HFA-treated specimens had a slightly rougher surface with more pronounced peaks and valleys. Additionally, the features in the PA-treated specimens were more densely packed and less evenly distributed. Overall, the milled specimens, especially those treated with HFA, exhibited the highest level of surface irregularities.

## Discussion

PCs most often fail to withstand the forces of orthodontic biomechanics. Treatment of bonding surfaces, either by chemical or mechanical methods, enhances the durability of the bonding to the PC. The current study investigated the SBS of orthodontic brackets to 3D-printed and milled provisional materials, comparing the effects of two chemical surface treatments: HFA etching and PA etching. The mean SBS Values of 3D-printed materials showed that HFA etching resulted in significantly higher SBS values, compared to PA etching, which are in line with other studies (14). In milled materials, no significant difference in SBS was observed between the two groups. Based on these results, the null hypothesis was rejected as there is a significant difference in the SBS of orthodontic brackets bonded to 3D-printed and milled PCs.

There were statistically significant differences between all groups except between the milled groups treated with HFA and PA (*p* = 0.916), indicating that in milled materials, the type of etching has less impact on the bonding strength. It was confirmed that 3D-printed materials generally offer better bonding conditions compared to milled ones. The results of the present study suggest that both the material and surface treatment can affect the SBS of orthodontic brackets favouring 3D-printed provisional material and HFA etching (*p* < 0.001). However, in the study by Ghozy et al., the type of CAD/CAM ceramic material did not affect SBS when tested with ceramic brackets (14).

The present study evaluated the amount of residual adhesive left on 3D-printed and milled materials after debonding using a digital microscope and reported the results using the ARI index. The 3D-printed models (3D-HFA and 3D-PA) demonstrate a wider distribution of scores (Score 0, 1, 2 and 3), indicating greater variation in bond strength. Our findings suggest that 3D-printed materials treated with HFA exhibited more cohesive failures. In contrast, milled materials often had no remaining adhesive, especially the PA-treated ones, and there was a clustering of low scores (Score 0). This indicated that the adhesive failed at the bond interface. These findings are consistent with previous studies (4, 15, 16). The 3D-printed HFA-treated cylinders showed a higher frequency of ARI scores of 2 or 3 which suggests that a substantial amount of adhesive remained on the specimen surfaces after debonding. This indicates a greater tendency towards cohesive failure between the adhesive and the provisional material. It may be due to the fact that etching with HFA increases the surface area, which helps the adhesive to penetrate into the microchannels created (17). Almost all the milled specimen (except one) had score 0, indicating adhesive failure and the absence of adhesive on the provisional material after debonding. These differences could be attributed to variations in surface characteristics of the provisional material, material internal structure, or interactions of the chemicals used in the surface treatment. These findings are essential for clinical considerations, as they suggest that 3D-printed materials may provide better adhesive retention but could be challenging during the debonding process.

SEM analysis of the bond interface revealed that the 3D-printed specimens exhibited smoother surfaces. The HFA-treated specimens had a slightly rougher surface than the PA specimens with more pronounced peaks and valleys. HFA-treated milled specimens exhibited the highest level of surface irregularities (3, 15, 18).

HFA surface treatment is known to enhance the strength of both 3D-printed and milled materials. However, its effectiveness in improving the bond strength of orthodontic brackets to CAD/CAM PCs has been debated (15, 19). A combination of chemical and mechanical treatments can enhance bond strength (20). Factors like the type of adhesive and aging through thermocycling can also impact bond strength (21). HFA creates silicon-oxygen (Si-O) bonds by dissolving silica-based filler particles in the 3D-printed material. This etching exposes reactive sites on the resin matrix, allowing for better adhesion of the adhesive through covalent bonding with silane coupling agents (22).

When an adhesive is applied, it penetrates into the surface irregularities and chemically bonds to the functional groups (like hydroxyl or ester groups) exposed by the etching process. The microstructure of milled crowns is more homogeneous and less porous compared to 3D-printed crowns. The etched surface of PMMA may appear rougher, but its bulk structure does not support deep penetration of the adhesive. In contrast, the 3D-printed materials have a more complex microstructure with silica fillers that enhance both surface roughness and chemical bonding potential at the interface. Additionally, the interface in 3D-printed crowns treated with HFA includes etched silica particles and resin matrix, offering both micro retention and chemical bonding sites for the adhesive. The milled crowns lack these features, resulting in weaker bonding.

The limitations of the study include that only two types of acid etching (HFA and PA) were investigated, and other surface treatments that might affect bond strength were not included in this study. Although thermocycling was performed to simulate oral conditions, the aging process is simplified. It may not represent the full range of stresses and environmental factors encountered in the mouth over time. Only one type of adhesive (Transbond XT) was used in bonding the brackets, which may not reflect the performance of other adhesives in clinical practice.

Future studies could include other types of provisional and definitive crown materials, as well as different 3D printing resins, to evaluate the bonding effectiveness across a wider range of materials. Conducting clinical trials would help validate the findings in real-world scenarios by assessing the long-term durability and effectiveness of bracket bonding under actual oral conditions. More complex aging processes, such as prolonged thermocycling, mechanical fatigue testing, and real-time chewing simulation, to better understand the durability of the bond over time in dynamic conditions can be included in future studies.

## Conclusion

Current research suggests that 3D-printed materials demonstrate superior SBS to orthodontic brackets compared to milled materials, indicating that they may provide a stronger bond. Further, HFA etching is more effective than PA etching in enhancing the SBS of orthodontic brackets to 3D-printed PC materials. However, for milled materials, the choice of etching agent has minimal impact on SBS.

## Data Availability

The original contributions presented in the study are included in the article/Supplementary Material, further inquiries can be directed to the corresponding author.
